# Complete Dosage Compensation in *Anopheles stephensi* and the Evolution of Sex-Biased Genes in Mosquitoes

**DOI:** 10.1093/gbe/evv115

**Published:** 2015-06-16

**Authors:** Xiaofang Jiang, James K. Biedler, Yumin Qi, Andrew Brantley Hall, Zhijian Tu

**Affiliations:** ^1^Program of Genetics, Bioinformatics, and Computational Biology, Virginia Tech, Blacksburg, Virginia; ^2^Department of Biochemistry, Virginia Tech, Blacksburg, Virginia

**Keywords:** comparative transcriptomes, RNA-seq, sex-specific expression, gene duplication

## Abstract

Complete dosage compensation refers to hyperexpression of the entire X or Z chromosome in organisms with heterogametic sex chromosomes (XY male or ZW female) in order to compensate for having only one copy of the X or Z chromosome. Recent analyses suggest that complete dosage compensation, as in *Drosophila melanogaster*, may not be the norm. There has been no systematic study focusing on dosage compensation in mosquitoes. However, analysis of dosage compensation in *Anopheles* mosquitoes provides opportunities for evolutionary insights, as the X chromosome of *Anopheles* and that of its Dipteran relative, *D. melanogaster* formed independently from the same ancestral chromosome. Furthermore, *Culicinae* mosquitoes, including the *Aedes* genus, have homomorphic sex-determining chromosomes, negating the need for dosage compensation. Thus, *Culicinae *genes provide a rare phylogenetic context to investigate dosage compensation in *Anopheles* mosquitoes. Here, we performed RNA-seq analysis of male and female samples of the Asian malaria mosquito *Anopheles stephensi* and the yellow fever mosquito *Aedes aegypti*. Autosomal and X-linked genes in *An. stephensi* showed very similar levels of expression in both males and females*,* indicating complete dosage compensation. The uniformity of average expression levels of autosomal and X-linked genes remained when *An. stephensi* gene expression was normalized by that of their *Ae. aegypti* orthologs, strengthening the finding of complete dosage compensation in *Anopheles*. In addition, we comparatively analyzed the differentially expressed genes between adult males and adult females in both species, investigated sex-biased gene chromosomal distribution patterns in *An. stephensi* and provided three examples where gene duplications may have enabled the acquisition of sex-specific expression during mosquito evolution.

## Introduction

Complete dosage compensation is a mechanism hypothesized to compensate for the loss of one copy of the X/Z chromosome in organisms with heterogametic sex chromosomes by hyperexpressing the entire X/Z chromosome (Ohno 1967; Mank 2013). Although complete dosage compensation has been demonstrated in model organisms such as *Drosophila melanogaster* (Straub and Becker 2007; Gelbart and Kuroda 2009), recent transcriptome analyses showed that dosage compensation is highly variable across species(Mank et al. 2011). Although a lack of complete dosage compensation for ZW chromosomes is observed in birds, blood flukes, and snakes (Mank and Ellegren 2008; Vicoso and Bachtrog 2011; Uebbing et al. 2013; Vicoso et al. 2013), there are also cases where ZW chromosomes displayed dosage compensation (Smith et al. 2014). In addition, there have been new challenges to the earlier conclusion that the eutherian X chromosome exhibits complete dosage compensation (Xiong et al. 2010; Deng et al. 2011; Lin et al. 2012). Analysis based on gene expression in patients with X aneuploidy syndrome showed that dosage-sensitive genes are dosage compensated but the remainder of X-linked genes may not be (Pessia et al. 2012; Wright and Mank 2012). It has also been recently demonstrated that complete dosage compensation does not always mean the same level of gene expression in males and females. X-linked genes in male flour beetles are hyperexpressed and on average reached the same expression levels as autosomal genes. X-linked genes in female flour beetles are also hyperexpressed resulting in a higher level of expression of X chromosome genes compared with males (Prince et al. 2010). However, the hyperexpression of the X chromosome in *Tribolium *females has recently been challenged (Mahajan and Bachtrog 2015). All these recent analyses make it clear that the mechanism of dosage compensation employed by one species may not be readily extrapolated to other species because dosage compensation evolves in concert with the formation of heteromorphic sex chromosomes, which could have independent origins and turn over rapidly (Mank et al. 2011).

The commonly accepted method to determine whether dosage compensation exists is to compare the overall expression level for X- or Z-linked genes to the overall expression level of autosomal genes in the heterogametic sex (Mank 2013). If the overall expression levels do not differ significantly, it can be assumed that complete dosage compensation exists. This method assumes that the overall proto-X/proto-Z chromosome expression level is and remains the same as the overall expression level of autosomes. However, these assumptions are not always valid. Proto-X/proto-Z chromosomes do not necessarily have the same overall expression level as other autosomes because the gene content and overall expression patterns differ between individual chromosomes. In addition, this method can be influenced by data processing and choice of statistical analysis if the number of lowly expressed genes differs between the X and autosome. A study of dosage compensation in placental mammals has been criticized for not filtering genes with low or no expression (Xiong et al. 2010; Deng et al. 2011; Kharchenko et al. 2011). The conclusions of the aforementioned study can differ based on different filtering criteria (Jue et al. 2013).

A more reliable test for dosage compensation is to compare the expression of X/Z-linked genes to that of their autosomal or pseudoautosomal orthologs in related species. This approach has been applied in mammals (Lin et al. 2012) and in *Drosophila pseudoobscura,* which has a neo-X chromosome (Nozawa et al. 2014).

Incomplete dosage compensation likely has caused the overrepresentation of sex-biased genes on the X/Z chromosomes in several species (Harrison et al. 2012; Uebbing et al. 2013). In some species with complete dosage compensation, sex-biased genes are not randomly distributed among the sex chromosomes and autosomes. In *Drosophila,* male-biased genes are underrepresented on the X chromosome (Vicoso and Charlesworth 2009; Magnusson et al. 2012). Several different hypotheses have been proposed to explain the paucity of X-linked male-biased genes including: sexual antagonism, the effects of dosage compensation, and male meiotic sex chromosome inactivation (Meiklejohn et al. 2011).

Dosage compensation and the chromosomal distribution of sex-biased genes are well-characterized in *D. melanogaster* (Larschan et al. 2011; Magnusson et al. 2012). A recent publication showed that dosage compensation evolved multiple times, consistently through upregulation of the single X in males during the numerous transitions of sex chromosomes in diverse fly taxa (Vicoso and Bachtrog 2015). However, these aspects of evolution have not been extensively studied in *Anopheles* mosquitoes, which belong to the same Dipteran order as *Drosophila,* but independently acquired the X chromosome (Toups and Hahn 2010; Pease and Hahn 2012). Unlike *Anopheles*, *Culicinae* mosquitoes including the *Aedes* and *Culex* genera, have a homomorphic sex-determining chromosome with pseudoautosomal regions spanning almost the entire length (Kitzmiller 1963; Hunter and Hartberg 1986). Combined with research based on retrogene movement, it has been hypothesized that the ancestor of *Anopheles *and *Culicinae* mosquitoes lacked heteromorphic sex chromosomes (Toups and Hahn 2010). After the *Culicinae-Anophelinae* divergence, approximately 150 Ma, heteromorphic X and Y chromosomes formed in *Anopheles* mosquitoes as the nonrecombining region around the male-determining locus of the proto-Y expanded (Toups and Hahn 2010). The *Anopheles *X chromosome has persisted for approximately 100 Myr and is present in all extant *Anopheles *species (Neafsey et al. 2015). In *Aedes aegypti*, male-determining gene is located on chromosome 1 (Hall et al. 2015). The p arm of chromosome 1 is mostly orthologous to X of *Anopheles stephensi,* and the q arm of chromosome 1 is mostly orthologous to part of 2R of *An. stephensi* (Nene et al. 2007).

Gene duplication is one common way to generate sex-biased genes (Parsch and Ellegren 2013). Genes can be duplicated through tandem duplication and retrotransposition. Following duplication, the expression pattern of the ancestral gene may remain unchanged, whereas the new duplicate can evolve sex-biased expression. Alternatively, the ancestral one may become specialized to one sex, whereas the new duplicate becomes biased to the other sex. Examples where genes obtained sex-biased expression postduplication to resolve sex conflicts have only been reported in *Drosophila* and *mice* (Connallon and Clark 2011; Gallach and Betrán 2011; Chen et al. 2012).

Here, we sequenced adult male and female whole body transcriptomes of the Asian malaria mosquito *An. stephensi* and the yellow fever mosquito *Ae. aegypti *(Nene et al. 2007). First, we evaluated the X-to-autosome expression ratio in *An. stephensi *with different filtering criteria to access dosage compensation. Then we performed comparisons with the *Aedes* diploid orthologs of X-linked genes in *An. stephensi* to investigate *Anophelinae* dosage compensation with phylogenetic context. In addition, we also performed comparative analysis of the sex-biased genes in the two species. We have also identified several examples where gene duplication may have enabled the acquisition of sex-specific expression during mosquito evolution.

## Materials and Methods

### RNA Isolation and RNA Sequencing

Mosquitoes that emerged over a 24-h period were either directly collected as 0- to 1-day-old adults, or isolated for later collection time points of 1- to 2-day-old and 2- to 3-day-old adults. For each time point, five mosquitoes were homogenized in 300 µl RNA lysis buffer (Zymo Research) and stored at −80 °C until RNA isolation. For RNA isolation, equal volumes of homogenate from each time point were combined to represent 0- to 3-day-old mosquitoes. These steps were performed in biological triplicates for males and virgin nonblood-feed females of both *An. stephensi *and *Ae. aegypti.* Illumina paired-end libraries for the resulting samples were prepared using the manufacturer’s specific protocol. The libraries were then sequenced using Illumina HiSeq. The resulting samples have been submitted to the NCBI SRA under the accession SRP047470 and SRP055921.

### Orthology and Chromosome Assignment

Orthology information was obtained from orthoDB (Waterhouse et al. 2013; http://cegg.unige.ch/orthodbmoz2, last accessed June 25, 2015). Genomic scaffolds and gene annotations for the *An. stephensi *Indian strain *and Ae. aegypti* were downloaded from VectorBase (https://www.vectorbase.org/, last accessed June 25, 2015). *Aedes aegypti* genome version 3.2 and annotation version 3.2, *An. stephensi* genome version 2 and annotation 2.2 were used. Based on the one-to-one ortholog pairs between *An. stephensi* and *An. gambiae*, *An. stephensi* scaffolds were assigned to chromosome arms. Assignment requires that each scaffold has at least three genes, more than 92% of the genes on the scaffold are orthologous to the same chromosome element (Neafsey et al. 2015), and no more than three contiguous genes are orthologous to other chromosome elements. Chromosomal assignments based on these criteria are reliable in *Anopheles* because there is no large-scale interchromosomal gene movement during *Anopheles* evolution (Neafsey et al. 2015). In total, 190 scaffolds containing 11,413 of 12,350 total genes were assigned to chromosome arms. Previous publications reported physical mapping of 60% of the *An. stephensi* Indian strain genome (Jiang et al. 2014). All 155 gene-containing scaffolds based on the reported physical mapping were included and were in complete agreement with our orthology-based chromosomal assignments.

### Dosage Compensation Analysis in *An*. stephensi

RNA-seq reads from triplicate male and female samples for both *An. stephensi *and *Ae. aegypti* were trimmed using trimmomatic (Bolger et al. 2014) with parameter “LEADING:3 TRAILING:3 SLIDINGWINDOW:4:15 MINLEN:36” and then aligned to their respective genomes using Tophat2 (Kim et al. 2013). Read counts for each gene based on the six samples were generated using HTSeq (Anders et al. 2014). We normalized the read count table through the RPKM (Reads per kb of sequence, per million mapped reads; Mortazavi et al. 2008) approach to estimate expression level. Normalization was performed with the TMM (trimmed mean of M-values) method in the R package edgeR (Robinson et al. 2011). Read counts for genes were used to calculate the Spearman’s correlation coefficient between samples. Because the replicates of the same sex were highly correlated (supplementary table S1, Supplementary Material online), the triplicate RNA-seq data from each sex were combined as one single fastq file and the average RPKM values from the combined files were used for the following analysis.

Inactive genes (RPKM = 0 in both sample) and genes with low expression levels (0 <RPKM<cutoff value) were removed from the analysis. Different cutoff values including 1–4 RPKM were used to define genes with low expression levels. The ratios of the median RPKM value of X-linked genes to the median RPKM value of autosomal genes in both males and females were calculated and used to assess whether dosage compensation is present in *An. stephensi*. The analyses were performed on unfiltered data sets as well as filtered data sets to explore how filtering and filtering with different criteria affected the analysis. Two-sample Wilcoxon rank sum tests were applied to test the overall difference between X-linked and autosomal gene expression level.

One-to-one ortholog pairs of *An. stephensi* and *Ae. aegypti* were generated from orthoDB (Waterhouse et al. 2013). Of the 7,236 ortholog pairs, 7,035 were assigned to chromosome arms in *An. stephensi*. Genes with RPKM values less than 2 were removed in both species, leaving 5,096 ortholog pairs. The Spearman’s correlations of the RPKM values between one-to-one ortholog pairs were examined (supplementary table S2, Supplementary Material online). The ratio of *An. stephensi* gene RPKM value to their *Ae. aegypti* ortholog RPKM value were calculated for each ortholog pair. The ratios were linearly adjusted by the same factor to make the median expression levels of *An. *stephensi autosomal genes the same as the median expression levels of their orthologs.

### Sex-Biased Gene Analysis

Three commonly used tools: CuffDiff (Trapnell et al. 2012), DESeq2 (Love et al. 2014), and edgeR (Robinson et al. 2010), were used for statistical analysis of differential expression between males and females. Read count tables for each triplicate male and female sample were used as input for both edgeR and DESeq2. The Tophat output from our previous analysis was further processed by Cufflinks and then differentially expressed genes between males and females were identified by CuffDiff. Genes that were detected as differentially expressed by at least two of the three metrics (FDR < 0.05 for edgeR, *P* value < 0.05 for cuffdiff, *q* value < 0.05 for DESeq2) were used as the final set of sex-biased genes in our analysis. The analysis was performed using the triplicate RNA-seq data from *An. stephensi* and *Ae. aegypti.*

The expression level bias between two sexes for individual genes was estimated by the magnitude of the difference in expression levels between the sexes. Sex-biased genes were divided into groups for further analysis based on the magnitude of the difference in the expression levels between the sexes. All protein sequences of *An. stephensi *and *Ae. aegypti *were used as input for BLAST2GO (Conesa et al. 2005) to retrieve GO (Gene Ontology) term information. Overrepresented GO terms for each group were identified using a hypergeometric test using the GOstats package (Beißbarth and Speed 2004) in R.

### Phylogenetic Inference

*Culicidae* orthologous protein sequences of the genes of interest were retrieved from orthoDB and fragmented sequences were removed manually. Sequences were aligned using MUSCLE (Edgar 2004) with default parameters. Alignments were trimmed with trimAl (Capella-Gutiérrez et al. 2009) with the parameter “-gt 0.8” to exclude alignment columns with gaps presented in more than 20% of the sequence. The trimmed alignments were used as input for MrBayes (Huelsenbeck 2001), a program for Bayesian estimation of phylogeny. The rate matrix for amino acid data was set as “mixed” for MrBayes analysis. MrBayes performed a Markov Chain Monte Carlo analysis for 1,000,000 generations with four chains with the temperature set to 0.2. The resulting consensus tree was visualized with FigTree (Version 1.4.2, http://tree.bio.ed.ac.uk/software/figtree, last accessed June 26, 2015). Phylogenetic trees were built to infer the relative time of duplication with respect to speciation of mosquitoes. The trees also assisted to distinguish the ancestral gene and derived duplicates.

## Results

### Complete Dosage Compensation in *An. stephensi* as Shown by the X-to-Autosome Expression Ratio

Based on the chromosomal location of *An. gambiae* orthologs, 190 *An. stephensi *scaffolds were assigned to chromosome arms. In total, 1,029 genes were assigned to the X chromosome, and 9,933 genes were assigned to autosomes (supplementary table S3, Supplementary Material online). Gene expression levels in RPKM were estimated from triplicate male and female RNA-seq samples. Correlations between samples of the same sex were statistically significant for both males and females (Spearman’s correlation >0.95). Thus, an average expression level was used for comparisons between the two sexes.

The most common method to determine whether dosage compensation is present is to compare the overall expression level of genes on the X/Z chromosome to that of autosomal genes in the heterogametic sex. In *An. stephensi*, this is the X-to-autosome (X:AA) ratio of gene expression in males. Genes with low or no expression can have large effects on the X:AA ratio of gene expression. Consequently, we removed genes with RPKM values below various arbitrary cutoffs and then used the median RPKM values for the X-linked and autosomal genes to derive the X:AA ratio. If there is complete dosage compensation, we expect the X:AA ratio to be approximately 1. In theory, when there is not dosage compensation, the X:AA ratio should be 0.5. However, due to the buffering effects of gene regulatory networks, even without complete dosage compensation the ratio could be 0.6–0.75 (Mank 2009; Harrison et al. 2012; Wright et al. 2012).

In males, no matter the filtering criteria used, the X:AA ratio was always greater than 0.94 indicating that there was complete dosage compensation. We used the Wilcoxon rank sum test to evaluate whether there was a statistical difference between the expression level of X-linked and autosomal genes. We found no statistical difference between the expression level of X-linked and autosomal genes when no filtering or filtering greater than 2 RPKM was applied (table 1 and supplementary table S4, Supplementary Material online).

**Table 1:** Effect of the Stringency of the Expression Level Cutoff on the Median Gene Expression of the X Chromosome and Autosomes

	Number of Genes Remained		Female				Male			
	X	Autosomes	X RPKM	Autosome RPKM	*P* Value*	XX:AA Ratio	X RPKM	Auto-some RPKM	*P* Value*	X:AA Ratio
**Original**	1,029	9,933	10.67	11.49	0.48	0.93	10.91	11.23	0.10	0.97
**Remove genes = 0 RPKM**	1,012	9,719	11.11	12.04	0.32	0.92	11.29	11.79	0.05	0.96
**Remove genes < 1 RPKM**	927	8,901	13.41	14.39	0.20	0.93	13.16	13.82	0.03	0.95
**Remove genes < 2 RPKM**	869	8,440	15.42	16.03	0.33	0.96	14.61	15.18	0.08	0.96
**Remove genes < 3 RPKM**	826	8,031	16.93	17.77	0.25	0.95	15.69	16.63	0.08	0.94
**Remove genes < 4 RPKM**	785	7,680	18.78	19.23	0.30	0.98	16.91	17.81	0.12	0.95

*P values were calculated based on two-sample Wilcoxon rank sum tests.

**Fig. 1.—:**
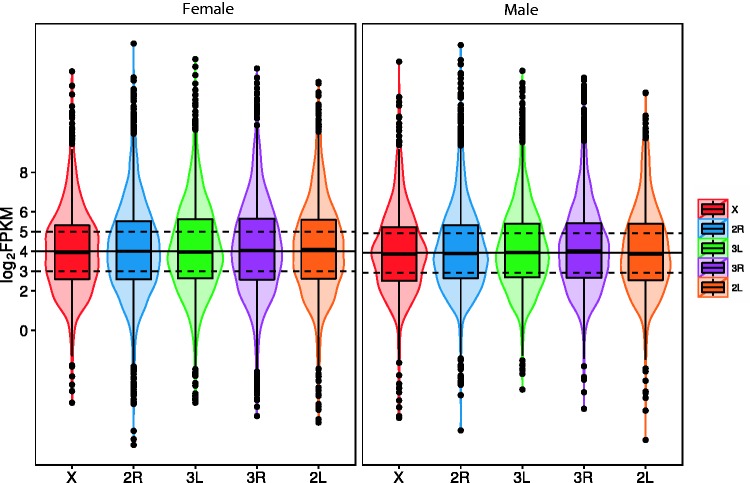
The distribution of log2 transformed RPKM values of genes on different autosomal arms and the X chromosome in males and females. Inactive and low-expressed genes (genes with RPKM value <2 in one of the samples) were removed in this analysis. The width of the violin plots shows the density of genes at different log2 RPKM values. Boxplots are also shown in which the bottom and top of the box are the first and third quartiles, and the solid band inside the box is the median. The solid black horizontal line in each panel represents the median log2 RPKM value of autosomes in the corresponding sample. Dashed black horizontal lines above and below the black lines represent +1 and −1 of median log2 RPKM.

We chose 2 as the RPKM cutoff for the analysis so that there was enough filtering stringency to remove noise and at the same time retain the maximum number of genes (fig. 1). We also performed the same analysis on individual replicates of male and female RNA-seq samples and observed similar results (supplementary fig. S1, Supplementary Material online). Taken together, these results strongly suggest there is complete dosage compensation in *An. stephensi.*

### Complete Dosage Compensation in *An. stephensi* as Shown by the Expression Ratio of *An. stephensi* X-Linked Genes to Their *Ae. aegypti* Orthologs

Although many studies have used the X:AA (or Z:AA) ratio to assess the presence of dosage compensation, concerns have been brought forth (Mank 2013). X-linked genes are not homologous to autosomal genes so the difference in gene content may result in differences in chromosome-wide gene expression levels independent of dosage compensation. These problems can be mitigated by comparing the expression levels of X/Z-linked genes to those of their autosomal or pseudoautosomal orthologs in related species. Here, we calculated the ratio of the expression level of X-linked genes in *An. stephensi* to their one-to-one orthologs in *Ae. aegypti* (X:XX expression ratio) as well as the expression level of autosomal genes in *An. stephensi* to their one-to-one orthologs in *Ae. aegypti* (AA:AA ) to assess whether dosage compensation is present in *An. stephensi*.

Orthologous genes may have different lengths between species, so we used normalized RPKM values. Genes with RPKM values less than 2 were removed. In total, 5,096 orthologous gene pairs were identified including 421 X-linked genes in *An. stephensi*, providing ample data for our analysis. The overall expression levels of one-to-one orthologs are strongly correlated (supplementary table S2, Supplementary Material online), with a correlation of 0.52 from female RNA-seq and 0.64 from male RNA-seq. The correlation is lower for X-linked genes compared with autosomal genes in both males (0.59) and females (0.48) but is still correlated. These lower correlation values may be a result of more adaptive selection pressure acting on the X chromosome causing higher X-linked divergence (faster-X effect) as has been observed in embryos and adult *Drosophila* (Kayserili et al. 2012)*.*

**Fig. 2.—:**
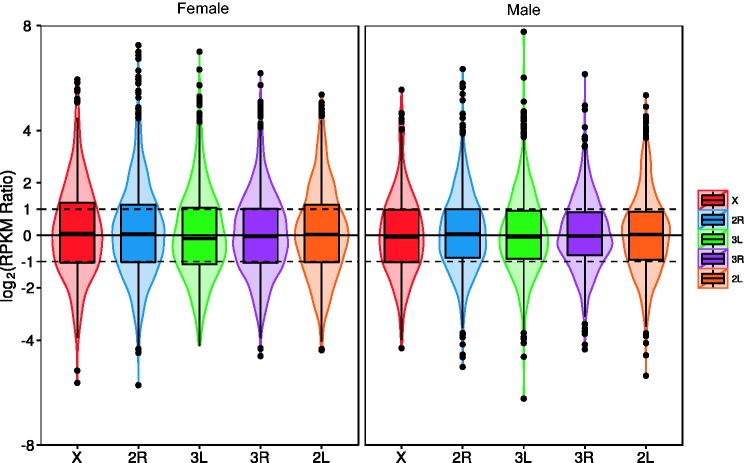
The distribution of the log2 normalized ratio of RPKM values in *An. stephensi* to their one-to-one orthologs in *Ae. aegypti* on different chromosome arms in males and females. The width of the violin plots shows the density of genes at different log2 RPKM ratios. Boxplots are also shown in which the bottom and top of the box are the first and third quartiles, and the solid band inside the box is the median. The solid black horizontal line in each panel represents 0 in the corresponding sample. The Dashed black horizontal lines above and below the black line represent +1 and −1.

We calculated the RPKM ratio for each pair of orthologs in *An. stephensi *and *Ae. aegypti.* We normalized the median RPKM ratio of *An. stephensi *autosomal genes to their orthologs in *Ae. aegypti* to 1 to adjust for differences in the overall expression levels between the species. After normalization, we observed that the median RPKM ratio of *An. stephensi *X-linked genes to their orthologs in *Ae. aegypti *was close to 1 in both sexes (fig. 2). These results indicate that there is dosage compensation for the X chromosome in male *An. stephensi. *The female X-linked gene expression level remained the same indicating that the dosage compensation mechanism is either exclusive to males or has been repressed in females.

### Sex-Biased Genes in *An. stephensi* and Their Chromosomal Distribution

**Fig. 3.—:**
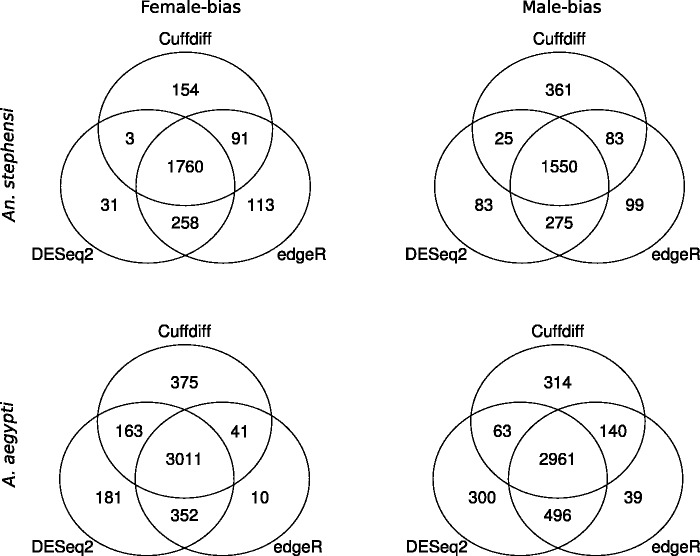
Venn diagrams of the overlap of differentially expressed genes based on Cufdiff, DESeq2, and edgeR.

We used three commonly used tools: Cuffdiff, DEseq2, and edgeR to identify differentially expressed genes between male and female samples in *An. stephensi*. We then used a Venn diagram to identify genes that were classified as differentially expressed using all three tools. Of the genes identified as differentially expressed by each method, more than 75% were identified as differentially expressed by the other methods (fig. 3). The overlap was greatest between DESeq2 and edgeR (2,018 for female-biased genes; 1,825 for male-biased genes), perhaps due to the similarity of the statistical approaches employed by these two methods. Here, we selected genes that were identified as differentially expressed by at least two methods for further analysis.

In *An. stephensi*, 2,112 genes were identified as female-biased and 1,933 genes were identified as male-biased. In *Ae. aegypti*, 3,567 genes were identified as female-biased and 3,660 genes were identified as male-biased. Ninety percent of female-biased genes and 82.7% of male-biased genes in *An. stephensi* have orthologs in *Ae. aegypti* (supplementary table S5, Supplementary Material online).

**Fig. 4.—:**
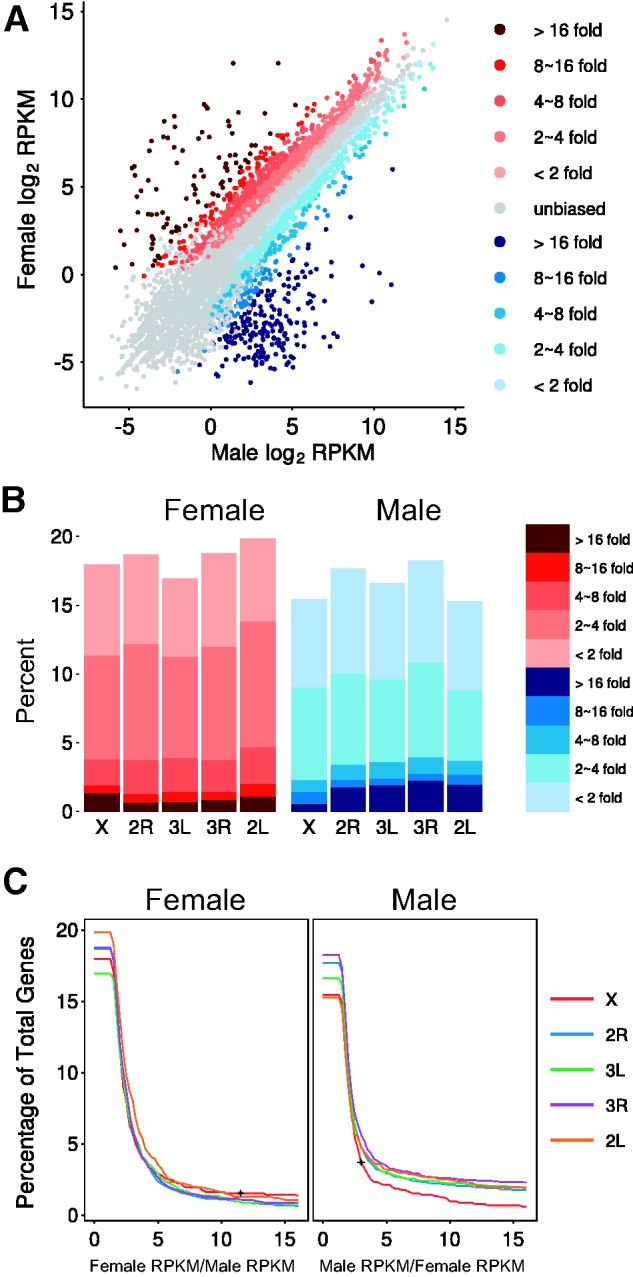
(*A*) Genome-wide sex-biased gene expression in *An. stephensi*. Darker shades of red represent greater female-biased expression. Darker shades of blue represent greater male-biased expression. (*B*) Percentage of sex-biased genes on five chromosomal arms. Left panel: female-biased genes; Right panel: male-biased genes. Darker shades of red represent greater female-biased expression. Darker shades of blue represent greater male-biased expression. (*C*) Percentage of total genes identified as sex-biased at different magnitudes of sex-bias between the sexes on individual chromosome arms. The *x* axis indicates female to male ratio (left panel, panel female) or male to female ratio (right panel, panel male) of gene expression levels. The asterisk in Female panel indicates the cutoff ratio (11.5) above which permutation tests showed X chromosome feminization. The asterisk in Male panel indicates the cutoff ratio (3) above which permutation tests showed X chromosome demasculinization.

We further categorized the sex-biased genes based on the magnitude of the difference in expression level between the sexes (fig. 4*A* and *B*). We then analyzed the GO terms enriched in the highly sex-biased genes. The most female-biased genes (log2 RPKM female to male ratio >4) were enriched for genes with molecular functions such as serine-type peptidase activity, proteolysis, and odorant binding, which is consistent with specialization of female mosquitoes for blood-feeding and subsequent blood-meal digestion. For the most part, male-biased genes (log2 RPKM male to female ratio >4) were overrepresented with GO terms such as microtubule-based movement, nucleosome assembly, and dynein complex, indicating involvement in spermatogenesis (supplementary file S1, Supplementary Material online).

Previous research has indicated that the distribution of sex-biased genes is nonrandom between sex chromosomes and autosomes (Parisi et al. 2003; Sturgill et al. 2007; Jaquiéry et al. 2013; Albritton et al. 2014). Magnusson et al. (2012) indicated demasculinization of the X chromosome in *An. gambiae* based on microarray data. To test whether the same pattern is observed in *An. stephensi* based on RNA-seq analysis, we investigated the chromosomal distribution of sex-biased genes.

When we set the threshold below a 3-fold difference between the sexes to define the term sex-biased, we observed no obvious demasculinization of the *An. stephensi* X chromosome (fig. 4*C*). However, when we increased the threshold to more than a 3-fold difference, we began to see an obvious demasculinization of the X chromosome similar to what was observed in *An. gambiae*. We observed both feminization and demasculinization of the X chromosome, when the threshold was greater than an 11.5-fold difference between males and females.

### Examples of Sex-Specific Subfunctionalization Post Gene Duplication

Sex-biased genes often arise from gene duplications. A gene that codes for the protein actin, *Actin-4,* has been well-characterized in *Ae. aegypti* and *Ae. albopictus* (Muñoz et al. 2004; Fu et al. 2010; Labbé et al. 2012). In *Ae. aegypti*, the *Actin-4* gene (*AAEL001951*) has two isoforms: the female isoform, which is highly expressed, and the male isoform, which is expressed at a lower level than the female isoform and codes for a nonfunctional protein. Our RNA-seq data show that *Actin-4* is female-biased in adults, and its paralog *Actin-3 *(*AAEL009451*) is male-biased (Vyazunova and Lan 2004). In *An. stephensi*, there are two *Actin-4* orthologs: *ASTEI10165,* which is extremely female-biased, and *ASTEI03074,* which is extremely male-biased (supplementary table S6, Supplementary Material online). We retrieved all available *Actin-4* orthologs from OrthoDB (Group MZ20123647) and built a phylogenetic tree (supplementary fig. S2, Supplementary Material online). As the tree shows, the duplication and subfunctionalization of this family of actin genes likely occurred before the divergence of *Aedes* and *Anopheles*.

A recent publication (Hall et al. 2014) identified a gene, *myo-sex*, which is tightly linked to the M-locus, is male-specific, and highly expressed in pupae of *Ae. aegypti*. *Myo-sex* and its paralog *AAEL005656 *likely originated from duplications of *AAEL005733* (Hall et al. 2014)*. *Based on our RNA-seq data, *myo-sex* is male-specific in adults and *AAEL005733* is male-biased with a 2-fold greater expression in adult males than in adult females. *AAEL005656* is extremely female-biased (supplementary table S6, Supplementary Material online). In *Anopheles*, only one ortholog of *AAEL005733 *(group MZ20123647) exists, suggesting there has not been a duplication. In *An. stephensi*, the ortholog of *AAEL005733 *is *ASTEI08310*. The expression pattern of *ASTEI08310* in both sexes is similar to that of *AAEL005733*, which may suggest that in *Ae. aegypti* the expression profile of the parental gene *AAEL005733* remains unchanged, whereas the two new copies specialized, each to a different sex.

Another example of duplicated genes becoming specialized in two sexes is the orthology group MZ22302531. Genes in this group are orthologs to venom allergens of wasps and fire ants, and belong to the family of cysteine-rich secretory proteins (Lu et al. 1993). This family is also related to mammalian testis-specific protein (Tpx-1), which is required for sperm capacitation (Kasahara et al. 1989). Based on RNA-seq data, the expression levels of paralogs of this group vary significantly between adult males and females. Of the four paralogs in *An. stephensi*, *ASTEI10265* is highly female-biased in adults, *ASTEI10266* is male-specific in adults, and the other two paralogs are barely expressed in adults (supplementary table S6, Supplementary Material online). In *Ae. aegypti*, in which there are six orthologs, *AAEL000793* is female-specific, *AAEL002693* is male-biased, *AAEL009239* is male-specific, and the rest are not significantly expressed in adults. We also checked the microarray data of *An. gambiae* on VectorBase. There are six paralogs in *An. gambiae*, and the genomic location indicates that all should have arisen as tandem duplications of the same ancestral gene. Also, the expression levels of these six paralogs vary between adult males and females: two are female-biased and two are male-biased. Based on phylogenic analysis (supplementary fig. S3, Supplementary Material online), the subfunctionalization of venom allergens occurred independently in *Aedes *and *Anopheles.*

## Discussion

### Dosage Compensation

We used RNA-seq to provide conclusive evidence that *An. stephensi *has complete dosage compensation. Dosage compensation is thought to evolve in concert with the formation of heteromorphic sex chromosomes (Charlesworth 1996; Mank 2013). Consequently, it is reasonable to assume that all *Anopheles *mosquitoes have dosage compensation and implement dosage compensation by the same or similar mechanisms because all *Anopheles *species share the same X chromosome (Neafsey et al. 2015). However, because the X chromosomes of *Anopheles *and *Drosophila* evolved independently (Toups and Hahn 2010), it is likely that their mechanisms for dosage compensation also evolved independently. In *Drosophila *species, dosage compensation is implemented by doubling the expression level of genes on the X chromosome in males (Conrad and Akhtar 2012). Dosage compensation in *Anopheles* could result from either the doubling of the expression level of X-linked genes in only males, or by doubling the expression level of X-linked genes in both males and females and subsequently silencing the expression of one X chromosome in females.

In *Drosophila*, the sex-lethal (*Sxl*) gene controls dosage compensation via male-specific lethal-2 (MLS2) through the (male-specific lethal) MSL-complex (Penalva and Sanchez 2003). Besides MSL-2, the MSL-complex includes males absent on the first (MOF), MSL1, MSL3, maleless (MLE), and the roX1 or roX2 noncoding RNAs (Conrad and Akhtar 2012). Even though some orthologs of these genes exist in some of the *Anopheles* genomes, these genes may perform different functions in *Anopheles* (Zdobnov 2002; Behura et al. 2011). For example, the ortholog of *Sxl* in *Anopheles* mosquitoes is not sex-specifically alternatively spliced and does not function in sex determination or the initiation of dosage compensation (Traut et al. 2006). However, because dosage compensation may evolve by modifying existing epigenetic regulation systems, research focused on conserved proteins involved in epigenetic networks may provide insights into the mechanism of dosage compensation in *Anopheles* (Graves 2014). Genes involved in the sex-determination pathway are often also involved in dosage compensation, such as in *Drosophila *species and *Bombyx mori* (Penalva and Sanchez 2003; Kiuchi et al. 2014).

*Drosophila *and *Anopheles* appear to have acquired dosage compensation independently. This example of convergent evolution may be attributed to shared features between these two families. First, both X chromosomes evolved from the same pair of autosomes, meaning that the same dosage-sensitive genes may have caused the need for dosage compensation in both species. Second, both families have relatively large effective population sizes. Large effective population size provides high genetic diversity, which may have led to a quick adaptive dosage compensation mechanism. Lastly, both genera are male heterozygotic. Although dosage compensation exists in ZW species (Smith et al. 2014), it is relatively more common in XY species due to several potential reasons: mutations occur more frequently in males, the X chromosome effective population size increases due to sexual selection, and stronger natural selection acts on males (Mank 2013).

### Sex-Biased Genes and Their Chromosomal Distribution

Sex-biased genes are generally identified through comparing male and female samples. Therefore, the number of sex-biased genes detected is dependent on factors such as: species, sampled tissue, and experimental and analytical methodology. Research on sex-biased genes in *An. gambiae* showed that because testes in males are proportionately smaller than ovaries in blood-fed females, testes-enriched genes were significantly underrepresented and ovary-enriched genes were highly overrepresented in the sex-biased genes detected from the whole body samples (Baker and Russell 2011; Baker et al. 2011). Here, we compare transcriptomes from adult male and adult virgin nonblood-fed female mosquitoes, which have much smaller ovaries than blood-fed females. Thus, the effect of allometry is not as pronounced as in comparison between males and blood-fed females. Future transcriptomic analysis of sex-, stage-, and tissue-specific samples of *An. stephensi* will enable the distinction between sex-biased genes detected in this research that result from overall sex-biased expression and those caused by tissue-specific expression.

Nonrandom distributions of sex-biased genes have been observed in several species (Vicoso and Bachtrog 2015). However, in 2005, Hahn and Lanzaro did not identify nonrandom distributions of sex-biased genes in *An. gambiae* (Hahn and Lanzaro 2005). Conversely, Magnusson et al. (2012) reported a deficit of male-biased genes in the *An. gambiae *X chromosome*.* Our data show that the X chromosome is depleted of genes with highly male-biased expression in *An. stephensi. *However, for genes with low sex-biased expression (<2-fold difference between the sexes), the trend does not exist. Highly male-biased genes are primarily expressed in the gonads, so the nonrandom distribution of highly male-biased genes may be attributed to specific tissues. This is consistent with GO terms associated with extremely male-biased genes (F/M > 16 or M/F > 16). This explanation is also consistent with the previous evidence of underrepresentation of testis-expressed genes on the *An. gambiae* X chromosome (Baker and Russell 2011). Further experiments like those done in several flies (Baker et al. 2011; Vicoso and Bachtrog 2015), where transcriptomes of adult female and male soma are sequenced will help to evaluate the contribution of gonads to the chromosomal distribution of sex-biased genes in *An. stephensi*.

### Gene Duplications in the Evolution of Sex-Biased Genes

We have shown that gene duplication is one mechanism that leads to the formation of new sex-biased genes and we present three examples of orthologous groups where duplicated gene copies become specialized, each to a different sex. Although the examples are identified from whole adult bodies, the sex-biased expression is likely due to tissue dimorphism. The sex-specific venom allergens are likely expressed in salivary glands based on previous research on their orthologs (Arcà et al. 2005), whereas the sex-specific genes actin and myosin are muscle-specific (Vyazunova and Lan 2004; Hall et al. 2014; Labbé et al. 2012). This is consistent with the findings that most of the genes that are specialized to one sex also become tissue-specific (Gallach and Betrán 2011; Chen et al. 2012; Wyman et al. 2012). As sexually antagonistic conflicts can be tissue-specific and stage-specific, duplicated genes can evolve specific expression profiles to solve the conflicts. Therefore, future transcriptomic studies on tissue- and stage-specific transcription in both males and females will provide a high resolution view of how sexually antagonistic conflicts affect mosquito gene expression and duplication.

*Actin-4* is expressed specifically in female flight muscles and has the highest level of expression during the pupal stage (Labbé et al. 2012). Our analysis shows that that *Actin-4* has female-biased expression in both adult *Ae. aegypti* and *An. stephensi*. The male-biased paralog of *Actin-4*, *Actin-3,* also has conserved expression in both species. *Actin-3* may perform a similar, but male-specific, function to *Actin-4*. Interestingly, despite the huge difference in the expression profiles, these two actin genes only differ by four amino acids, indicating that small mutations between these two genes may have large functional consequences. The interactions of actin and myosin are crucial for functions in the cell, including movement. Thus, actin and myosin genes may coevolve. The sex-specific subfunctionalization of the duplicated myosin genes in *Ae. aegypti* may have been partially triggered by the sex-specialization of actin genes. Nevertheless, more studies on the expression profiles and functional characterizations will further our understanding of these genes.

### Potential Vector Control Applications of Dosage Compensation and Sex-Specific Genes

Mosquitoes transmit pathogens to humans and livestock. For example, *An. gambiae* is the primary malaria vector in Africa and *An. stephensi* is the key vector of urban malaria on the Indian subcontinent (Singh et al. 1999). *Aedes aegypti* is a major vector of dengue fever, yellow fever, and *chikungunya* (Nene et al. 2007). Only female mosquitoes feed on blood and transmit disease, whereas males are harmless. Dosage compensation functions on a sex-to-sex basis. Thus, manipulation of genes involved in dosage compensation can potentially result in female lethality by distorting X-linked gene expression dosage. In addition, the study of sex-biased genes will shed light on mosquito sex determination and sexual differentiation, processes that can be used in novel genetic approaches for vector control. For example, the female-specific promoter of Actin-4 has been used to create flightless female mosquitoes as a vector control strategy (Fu et al. 2010; Labbé et al. 2012). Other sex-biased genes such as the myosin genes identified in this study could be used in genetic vector control strategies.

## Supplementary Material

Supplementary file S1, tables S1–S7, and figures S1–S3 are available at *Genome Biology and Evolution *online (http://www.gbe.oxfordjournals.org/).

Supplementary Data
