# Supplementary material for: Complete Dosage Compensation in Anopheles stephensi and the Evolution of Sex-Biased Genes in Mosquitoes
Source: Genome Biol Evol. 2015 Jun 16;7(7):1914–24. doi: 10.1093/gbe/evv115 (PMC4524482; doi:10.1093/gbe/evv115)
Supplement: Supplementary Data [file supp_7_7_1914__index.html]

Complete Dosage Compensation in Anopheles stephensi and the Evolution of Sex-Biased Genes in Mosquitoes — Supplementary Data 

# Complete Dosage Compensation in *Anopheles stephensi* and the Evolution of Sex-Biased Genes in Mosquitoes

## Supplementary Data

files

- Supplementary Data - xlsx file
- Supplementary Data - docx file
