# Supplementary material for: Complete Dosage Compensation in Anopheles stephensi and the Evolution of Sex-Biased Genes in Mosquitoes
Source: Genome Biol Evol. 2015 Jun 16;7(7):1914–24. doi: 10.1093/gbe/evv115 (PMC4524482; doi:10.1093/gbe/evv115)
Supplement: Supplementary Data [file supp_evv115_Supplementary_Data.docx]

Table S1

Spearman’s correlation between triplicate male and female RNA-seq samples in *An. stephensi.*

|  | **F1** | **F2** | **F3** | **M1** | **M2** | **M3** |
| --- | --- | --- | --- | --- | --- | --- |
| **F1** | 1.00 | 0.97 | 0.98 | 0.89 | 0.88 | 0.88 |
| **F2** | 0.97 | 1.00 | 0.98 | 0.88 | 0.85 | 0.86 |
| **F3** | 0.98 | 0.98 | 1.00 | 0.89 | 0.87 | 0.87 |
| **M1** | 0.89 | 0.88 | 0.89 | 1.00 | 0.96 | 0.97 |
| **M2** | 0.88 | 0.85 | 0.87 | 0.96 | 1.00 | 0.98 |
| **M3** | 0.88 | 0.86 | 0.87 | 0.97 | 0.98 | 1.00 |

Table S2

Comparison of the expression level of one-to-one orthologs on different chromosome arms.

| **Chromosomal arms** | **# of Pairs** | **Spearman’s correlation  between ortholog pairs** | | **Median of normalized *An. stephensi* to *A. aegypti* ratio** | |
| --- | --- | --- | --- | --- | --- |
|  |  | **Female** | **Male** | **Female** | **Male** |
| X | 421 | 0.48 | 0.59 | 1.05 | 0.96 |
| 2R | 1697 | 0.48 | 0.63 | 1.03 | 1.03 |
| 3L | 1050 | 0.53 | 0.64 | 0.92 | 0.96 |
| 3R | 1069 | 0.60 | 0.70 | 0.97 | 0.98 |
| 2L | 859 | 0.52 | 0.61 | 1.02 | 1.02 |
| Total | 5096 | 0.52 | 0.64 |  |  |

Table S3

Number of scaffolds and genes assigned to each chromosome arm.

|  | **X** | **2R** | **3L** | **3R** | **2L** | **Total** |
| --- | --- | --- | --- | --- | --- | --- |
| **Scaffolds** | 19 | 43 | 49 | 45 | 34 | 190 |
| **Genes** | 1029 | 3342 | 2558 | 2193 | 1840 | 10962 |

Table S4

The effect of stringency of the expression level cutoff on the median gene expression levels of individual chromosomal arms

|  | **Chromosomal arm** | **Original** | **Remove genes = 0 RPKM** | **Remove genes < 1 RPKM** | **Remove genes < 2 RPKM** | **Remove genes < 3 RPKM** | **Remove genes < 4 RPKM** |
| --- | --- | --- | --- | --- | --- | --- | --- |
| # of genes remaining | X | 1029 | 1012 | 927 | 869 | 826 | 785 |
|  | 2R | 3342 | 3273 | 3018 | 2863 | 2717 | 2615 |
|  | 3L | 2558 | 2497 | 2265 | 2139 | 2036 | 1958 |
|  | 3R | 2193 | 2157 | 1978 | 1901 | 1824 | 1724 |
|  | 2L | 1840 | 1792 | 1640 | 1537 | 1454 | 1383 |
| Female | X | 10.67 | 11.11 | 13.41 | 15.42 | 16.93 | 18.78 |
|  | 2R | 11.71 | 12.05 | 14.14 | 15.9 | 17.77 | 18.93 |
|  | 3L | 11.16 | 11.87 | 14.14 | 15.52 | 16.97 | 18.57 |
|  | 3R | 12.27 | 12.69 | 15.49 | 16.52 | 17.82 | 19.55 |
|  | 2L | 10.78 | 11.56 | 14.11 | 16.53 | 18.44 | 20.09 |
| Male | X | 10.91 | 11.29 | 13.16 | 14.61 | 15.69 | 16.91 |
|  | 2R | 11.31 | 11.75 | 13.38 | 14.77 | 15.97 | 17.05 |
|  | 3L | 11.21 | 11.96 | 13.98 | 15.38 | 16.92 | 17.83 |
|  | 3R | 12.02 | 12.53 | 15.06 | 16.32 | 17.27 | 18.85 |
|  | 2L | 10.02 | 10.79 | 12.67 | 14.52 | 16.22 | 17.88 |

Table S5

The number of genes in *An. stephensi* with the same expression pattern as their *A. aegypti* orthologs

|  | # of genes in the genome | Have *A. aegypti* orthologs | Same expression pattern as *A. aegypti* orthologs |
| --- | --- | --- | --- |
| Total genes | 11789 | 9415 | 5801 |
| Female-biased Genes | 2112 | 1900 | 919 |
| Male-biased Genes | 1933 | 1600 | 840 |
| Unbiased genes | 7744 | 5915 | 4042 |

Table S6

RPKM expression values of genes studied in the “examples of sex-specific subfunctionalization post gene duplication” section

|  | Female 1 | Female 2 | Female 3 | Male 1 | Male 2 | Male 3 |
| --- | --- | --- | --- | --- | --- | --- |
| AAEL001951 | 33.9 | 34.59 | 50.47 | 22.75 | 19.14 | 27.29 |
| AAEL009451 | 0.08 | 0.03 | 0.02 | 2.39 | 0.75 | 4.76 |
| ASTEI10165 | 4654.5 | 3862.56 | 4216.87 | 25.42 | 14.34 | 15.01 |
| ASTEI03074 | 1.73 | 0.85 | 0.7 | 1695.69 | 482.09 | 2659.16 |
| AAEL005656 | 148.08 | 141.29 | 193.5 | 0.27 | 0.47 | 0.39 |
| AAEL005733 | 432.12 | 450.81 | 477.79 | 928.08 | 1078.43 | 921.21 |
| ASTEI08310 | 624.52 | 413.77 | 492.52 | 724.29 | 576.74 | 1160.09 |
| AAEL000793 | 66.87 | 121.21 | 135.78 | 0.29 | 0.33 | 0.19 |
| AAEL002476 | 0.51 | 1.07 | 0.84 | 0.54 | 1.14 | 1.44 |
| AAEL002682 | 0 | 0 | 0 | 0 | 0.81 | 0.17 |
| AAEL002693 | 34.5 | 52.02 | 63.77 | 328.75 | 477.7 | 470.27 |
| AAEL006524 | 0 | 0.09 | 0.02 | 0.16 | 0.19 | 0.23 |
| AAEL009239 | 0.3 | 0.1 | 0.16 | 179.05 | 234.35 | 228.6 |
| ASTEI10265 | 243.91 | 303.25 | 252.83 | 2.8 | 2.2 | 2.5 |
| ASTEI10266 | 0 | 0 | 0 | 55.7 | 35.16 | 31.28 |
| ASTEI10267 | 0 | 0 | 0 | 0 | 0 | 0 |
| ASTEI02105 | 1.18 | 0 | 0.26 | 0 | 0 | 0 |

Table S7

Species information for the genes in the phylogentic tree (S Figure2 and S Figure3)

| **Actin (orthology group “MZ20123647”)** | |
| --- | --- |
| *Aedes aegypti* | AAEL001951, AAEL009451 |
| *Anopheles arabiensis* | AARA002120 |
| *Anopheles atroparvus* | AATE004853, AATE017801 |
| *Anopheles christyi* | ACHR005210, ACHR007823 |
| *Anopheles culicifacies* | ACUA026444 |
| *Anopheles darlingi* | ADAC008233 |
| *Anopheles dirus* | ADIR010427 |
| *Anopheles epiroticus* | AEPI001692 |
| *Anopheles funestus* | AFUN002506, AFUN002623 |
| *Anopheles gambiae* | AGAP001676 |
| *Anopheles melas* | AMEC010159, AMEM009124, AMEM017875 |
| *Anopheles quadriannulatus* | AQUA000539 |
| *Anopheles sinensis* | ASIS009415 |
| *Anopheles stephensi* | ASTEI03074, ASTEI10165 |
| *Culex quinquefasciatus* | CPIJ012090, CPIJ012572 |
|  | |
| **Allergen (ortholog group “MZ20138926”)** | |
| *Aedes aegypti* | AAEL000793, AAEL002476, AAEL002682, AAEL002693, AAEL006524, AAEL009239 |
| *Anopheles albimanus* | AALB004200, AALB005082, AALB005083, AALB005100 |
| *Anopheles arabiensis* | AARA002312, AARA008298, AARA008299, AARA008320 |
| *Anopheles atroparvus* | AATE003790, AATE010315, AATE019684, AATE020696 |
| *Anopheles christyi* | ACHR009278, ACHR009568, ACHR010244 |
| *Anopheles culicifacies* | ACUA004641, ACUA015427, ACUA016170 |
| *Anopheles darlingi* | ADAC003505, ADAC003516, ADAC003517, ADAC003518 |
| *Anopheles dirus* | ADIR007691, ADIR007693, ADIR008758 |
| *Anopheles epiroticus* | AEPI005724, AEPI005725, AEPI008397, AEPI010804, AEPI011424 |
| *Anopheles farauti* | AFAF001464, AFAF003020, AFAF020815, AFAF021038 |
| *Anopheles funestus* | AFUN007481, AFUN007671 |
| *Anopheles gambiae* | AGAP006417, AGAP006418, AGAP006419, AGAP006420, AGAP006421, AGAP006443 |
| *Anopheles maculatus* | AMAM002605, AMAM004433 |
| *Anopheles melas* | AMEC007890, AMEC010278, AMEC012577, AMEC020481, AMEC021084 |
| *Anopheles merus* | AMEM000805, AMEM002026, AMEM004964, AMEM007398, AMEM010880, AMEM017124, AMEM017902 |
| *Anopheles minimus* | AMIN007945, AMIN007946, AMIN007947, AMIN010824 |
| *Anopheles quadriannulatus* | AQUA006259, AQUA006260, AQUA006280, AQUA009698 |
| *Anopheles sinensis* | ASIS001015, ASIS002664, ASIS005129, ASIS005617, ASIS007445, ASIS010960, ASIS013026 |
| *Anopheles stephensi* | ASTEI02105, ASTEI10265, ASTEI10266, ASTEI10267 |
| *Culex quinquefasciatus* | CPIJ001849, CPIJ004028, CPIJ004029, CPIJ004030, CPIJ004031, CPIJ011834, CPIJ012293, CPIJ013075 |
| *Drosophila melanogaster* | FBgn0015010, FBgn0052679 |


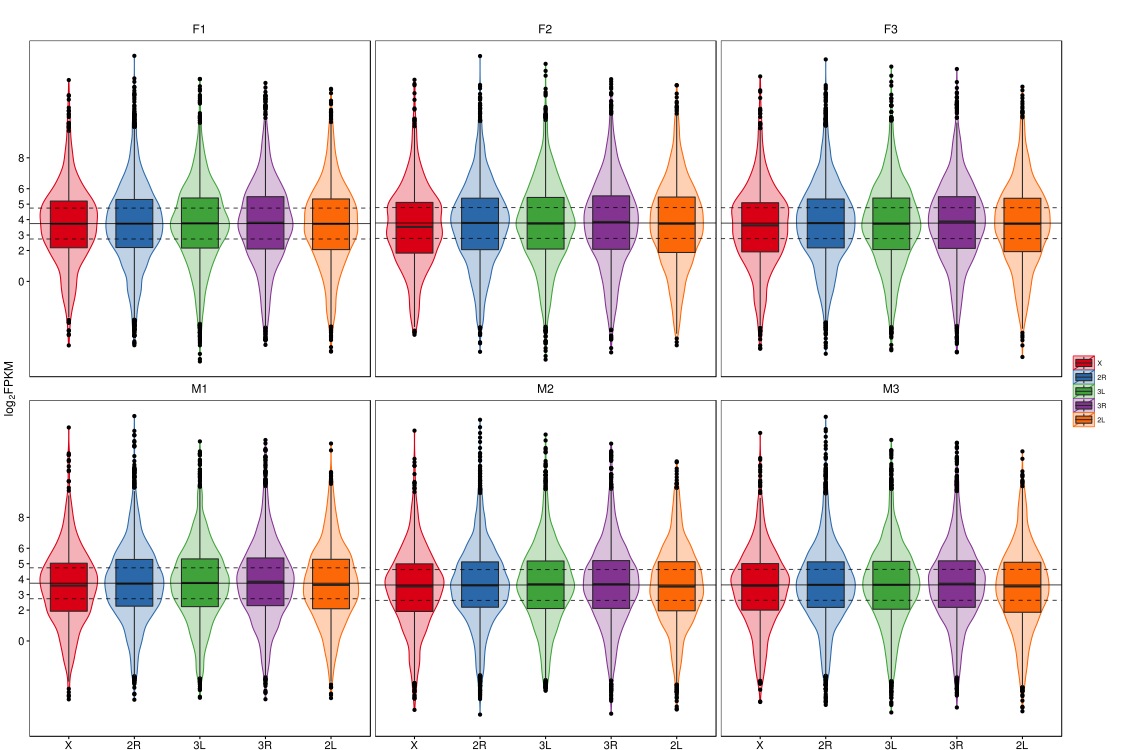


Figure S1. Distirbution of log2 transformed RPKM values of genes on different chromsome arms based on triplicate male (M1, M2, M3) RNA-seq samples and triplicate female (F1, F2, F3) RNA-seq samples. Inactive genes (genes with RPKM value equal to zero) were removed in this analysis. Violin plots show the density of the number of genes at different log2 RPKM values. For the boxplot, the bottom and top of the box are the first and third quartiles, and the band inside the box the median. The solid black horizonal line in each panel represents the median log2 RPKM value of autosomes in the corresponding sample. Dashed black horizonal lines above and below the black lines represent +1 and -1 of median log2 RPKM.

Figure S2. Phylogenetic tree of actins in *Culicidae.* The unrooted phylogenetic tree was generated using MrBayes 3.2. Clades are annotated with posterior probabilities. See Table S7 for gene information.

Figure S3. Phylogenetic tree of venom allergens in *Culicidae* with two *Drosophila meanogaster* orthologs (FBgn0052679 and FBgn0015010) as outgroups. Phylogenetic tree were generated using MrBayes 3.2. Clades are annotated with posterior probabilities. See Table S7 for gene information.
